# Comparative Transcriptomic Characterization of the Early Development in Pacific White Shrimp *Litopenaeus vannamei*

**DOI:** 10.1371/journal.pone.0106201

**Published:** 2014-09-08

**Authors:** Jiankai Wei, Xiaojun Zhang, Yang Yu, Hao Huang, Fuhua Li, Jianhai Xiang

**Affiliations:** 1 Key Laboratory of Experimental Marine Biology, Institute of Oceanology, Chinese Academy of Sciences, Qingdao, China; 2 University of Chinese Academy of Sciences, Beijing, China; 3 Hainan Guandtop Ocean Breeding Co. Ltd, Haikou, China; Chang Gung University, Taiwan

## Abstract

Penaeid shrimp has a distinctive metamorphosis stage during early development. Although morphological and biochemical studies about this ontogeny have been developed for decades, researches on gene expression level are still scarce. In this study, we have investigated the transcriptomes of five continuous developmental stages in Pacific white shrimp (*Litopenaeus vannamei*) with high throughput Illumina sequencing technology. The reads were assembled and clustered into 66,815 unigenes, of which 32,398 have putative homologues in nr database, 14,981 have been classified into diverse functional categories by Gene Ontology (GO) annotation and 26,257 have been associated with 255 pathways by KEGG pathway mapping. Meanwhile, the differentially expressed genes (DEGs) between adjacent developmental stages were identified and gene expression patterns were clustered. By GO term enrichment analysis, KEGG pathway enrichment analysis and functional gene profiling, the physiological changes during shrimp metamorphosis could be better understood, especially histogenesis, diet transition, muscle development and exoskeleton reconstruction. In conclusion, this is the first study that characterized the integrated transcriptomic profiles during early development of penaeid shrimp, and these findings will serve as significant references for shrimp developmental biology and aquaculture research.

## Introduction

**Figure 1:**
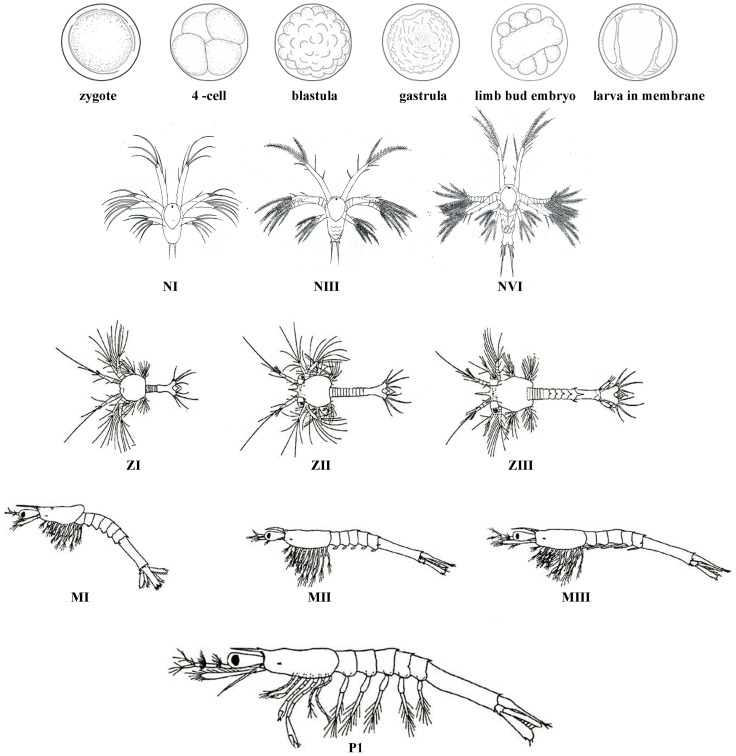
Embryonic and larval stages during early development of *L. vannamei.* (Z, M and P are modified from Hertzler PL, 2009). The developmental stages drawn in this figure include zygote, 4-cell, blastula, gastrula, limb bud embryo, larva in membrane, NI, NIII, NVI, ZI, ZII, ZIII, MI, MII, MIII and P1.

Pacific white shrimp (*Litopenaeus vannamei*) is one of the most economically important marine aquaculture species and farmed widespread over the world [1]. As a member of Crustacea, it has a distinctive pattern for early development by passing through embryo, nauplius, zoea, mysis and postlarvae [2]. In embryo stage, it gets through the journey from zygote to 2 cell, 4 cell, blastula, gastrula, limb bud embryo and larva in membrane. After hatching from membrane, it also experienced six nauplius stages, three zoea stages, three mysis stages and postlarvae stages before it becomes a juvenile shrimp (Figure 1). This pattern linked by metamorphosis is an important evolutionary and developmental transition and is a remarkable example of modularity in life cycles [3]. Both its morphological and physiological features change dramatically in this period, also leading to a high uncontrollability in larval rearing [4]. So the researches about early development of *L. vannamei* are of considerable significance for both developmental biology and aquaculture in penaeid shrimp.

During shrimp metamorphosis, physiological experiments are difficult to conduct due to the small size and rapid development. Up to now, the researches about early development of shrimp are mainly from morphological observation and biochemical analysis. Some studies focused on the impact of environmental factors on metamorphosis [4]–[6] in order to improve larvae survival rates in aquaculture [7]. Studies about the salinity and temperature optima for penaeid larvae have been performed in many species [4], [8], [9]. Some studies characterized enzyme activities which can be used for evaluating their physiological status. The activities of phenoloxidase, superoxide dismutase and peroxidase were measured as immunological parameters during ontology of *L. vannamei* [10]. Digestive enzyme activities which were closely connected with feeding habits also have been extensively studied such as trypsin and chymotrypsin [11], [12]. Nevertheless, functional genes related to early development are rarely reported. The molecular mechanisms of many important physiological changes in metamorphosis also have not been fully understood, and little is known about the dynamic change on gene expression level during early development.

Recently, the next generation sequencing (NGS) technology has flourished tremendously and is becoming an important method for measuring gene expression levels [13]. The advent of RNA-Seq provides a far more high-throughput and precise measurement of levels of transcripts and their isoforms than other methods [14]. It gives a general view of gene expression especially in these species lack of a fully sequenced and assembled genome such as *L. vannamei*. RNA-Seq has been reported in adult shrimp to identify immune related genes under WSSV or TSV infection [15]–[18]. The transcriptome of *L. vannamei* postlarvae (20 days post spawning) were also sequenced and annotated [19]. However, the transcriptional profiles across the early development for comparative analysis are still absent. The transition from comparing a few genes to whole transcriptomes is a vital approach for enhancing our understanding about this ontology [3].

In this study, we analyzed the transcriptomic characterization of *L. vannamei* during five different early development stages through Illumina high-throughput sequencing data. Results obtained from this study will contribute to further studies about molecular mechanisms for early development of *L. vannamei* and can be used for evolutionary analysis, developmental biology and functional gene research in penaeid shrimp.

## Materials and Methods

### Embryos and larvae sampling

The *L. vannamei* samples of different development stages were collected from Guangtai shrimp farm in Wenchang, Hainan, China. No specific permissions were required for the sampling locations and activities, and the studies did not involve endangered or protected species and locations. A total of 15 samples were collected based on their development stages: zygote, blastula, gastrula, limb bud embryo, larva in membrane, nauplius I (NI), nauplius III (NIII), nauplius VI (NVI), zoea I (ZI), zoea II (ZII), zoea III (ZIII), mysis I (MI), mysis II (MII), mysis III (MIII) and postlarvae 1 (P1). Each stage was identified according to observation with microscope. They were reared in a 25 m^3^ indoor pond with seawater at 31°C, salinity of 2.5%. They were unfed during embryo and nauplius stages. At zoea stage they were fed with spirulina and multiform formulated diet, while at mysis and postlarvae stages they were fed with artemia nauplii and multiform formulated diet. Embryos and larvae were collected with screen mesh at each stage when 90% of the population had reached the objective stage. Samples were immediately preserved in liquid nitrogen and then stored in −80°C for assays.

### RNA isolation and sample pooling

The total RNA of 15 samples was extracted separately by Unizol reagent (Biostar, China) following the manufacturer's instructions, RNA were assessed by electrophoresis in 1% agarose gel and quantified by NanoDrop 1000 spectrophotometer (Thermo Scientific, USA) and Agilent 2100 Bioanalyzer (Agilent Technologies, USA). Afterwards, the RNA samples of zygote, blastula, gastrula, limb bud embryo and larva in membrane were mixed equivalently into embryo sample (E), the RNA samples of NI, NIII and NVI were mixed equivalently into nauplius sample (N), the RNA samples of ZI, ZII and ZIII were mixed equivalently into zoea sample (Z), the RNA samples of MI, MII and MIII were mixed equivalently into mysis sample (M) and the RNA samples of postlarvae 1 were considered as postlarvae sample (P). The sample mixture was based on both morphological classification and physiological characters (Figure 1). Samples of zygote, blastula, gastrula, limb bud embryo and larva in membrane were typical stages before hatching. They mixed into E sample and represented the features of embryo in membrane. Similarly, NI, NIII and NVI composed N sample which represented nauplius stage, ZI, ZII and ZIII composed Z sample which represented zoea stage and MI, MII and MIII composed M sample which represented mysis stage. Then the five mixed RNA samples were used for library construction and sequencing.

### Library construction and Illumina sequencing

RNA purification, reverse transcription, library construction and sequencing were conducted by BGI (Shenzhen, China). To sum up, beads with Oligo(dT) were used to isolate and collect poly(A) mRNA from the mixed RNA. Fragmentation buffer was added for interrupting mRNA to short fragments. Using these short fragments as templates, random hexamer-primer was used to synthesize the first-strand cDNA. The second-strand cDNA was synthesized using buffer, dNTPs, RNase H and DNA polymerase I. Short fragments were purified with QiaQuick PCR extraction kit (Qiagen, Germany) and resolved with EB buffer for end reparation and tailing A. After that, the short fragments were connected with sequencing adapters. And, after the agarose gel electrophoresis, the suitable 200 bp fragment were selected for the PCR amplification as templates. At last, the libraries were sequenced using HiSeq 2000 (Illumina, USA).

### Sequencing data assembly and annotation

Image data from sequencing machine was transformed into raw reads by base calling, and stored in fastq format. The raw reads of all five samples were preprocessed by removing adaptors, reads with unknown nucleotides larger than 5% and low quality reads. The clean reads of each stage were then assembled into unigenes using the Trinity program [20]. Unigenes of five samples were then clustered into all-unigenes using TGICL [21]. In order to annotate all-unigenes, blast alignments [22] (E value < 1e-5) against the nr, nt, Swiss-Prot, KEGG, and COG databases were performed. Gene ontology (GO) analysis was carried out using BLAST2GO program [23].

### Analysis of differentially expressed unigenes

By means of reads mapping to all-unigenes, the FPKM (Fragments Per Kilo bases per Million fragments) value [24] of all-unigenes in each sample were obtained and used for comparing the expression difference between samples. Hierarchical clustering analysis (HCA) and principal components analysis (PCA) were performed using R [25]. We use FPKM value for comparing the expression difference between adjacent samples (E-N, N-Z, Z-M and M-P). We chose those with FDR (false discovery rate) ≤ 0.001 and absolute value of log2ratio ≥ 1 as differentially expressed genes (DEGs). Hypergeometric test was used to find significantly enriched GO terms and KEGG pathways in DEGs comparing to the whole background. After Bonferroni correction for p value, we defined corrected p value ≤ 0.05 as significantly enriched GO terms and KEGG pathways. The unigenes analyzed in this article for heat map were grouped together according to their FPKM values by Cluster 3.0 [26] and visualized by TreeView 1.6 [27].

### Validation by quantitative real-time PCR

Quantitative real-time PCR (qPCR) analysis was used for validation. 18S rRNA gene was used as an internal standard and relative gene expression levels were calculated using the comparative Ct method with the formula 2^−ΔΔCt^ [28]. The qPCR results were then compared with transcriptome data (FPKM value) to detect their expression correlation of each gene.

## Results and Discussion

### Illumina sequencing and *de novo* assembly

**Figure 2:**
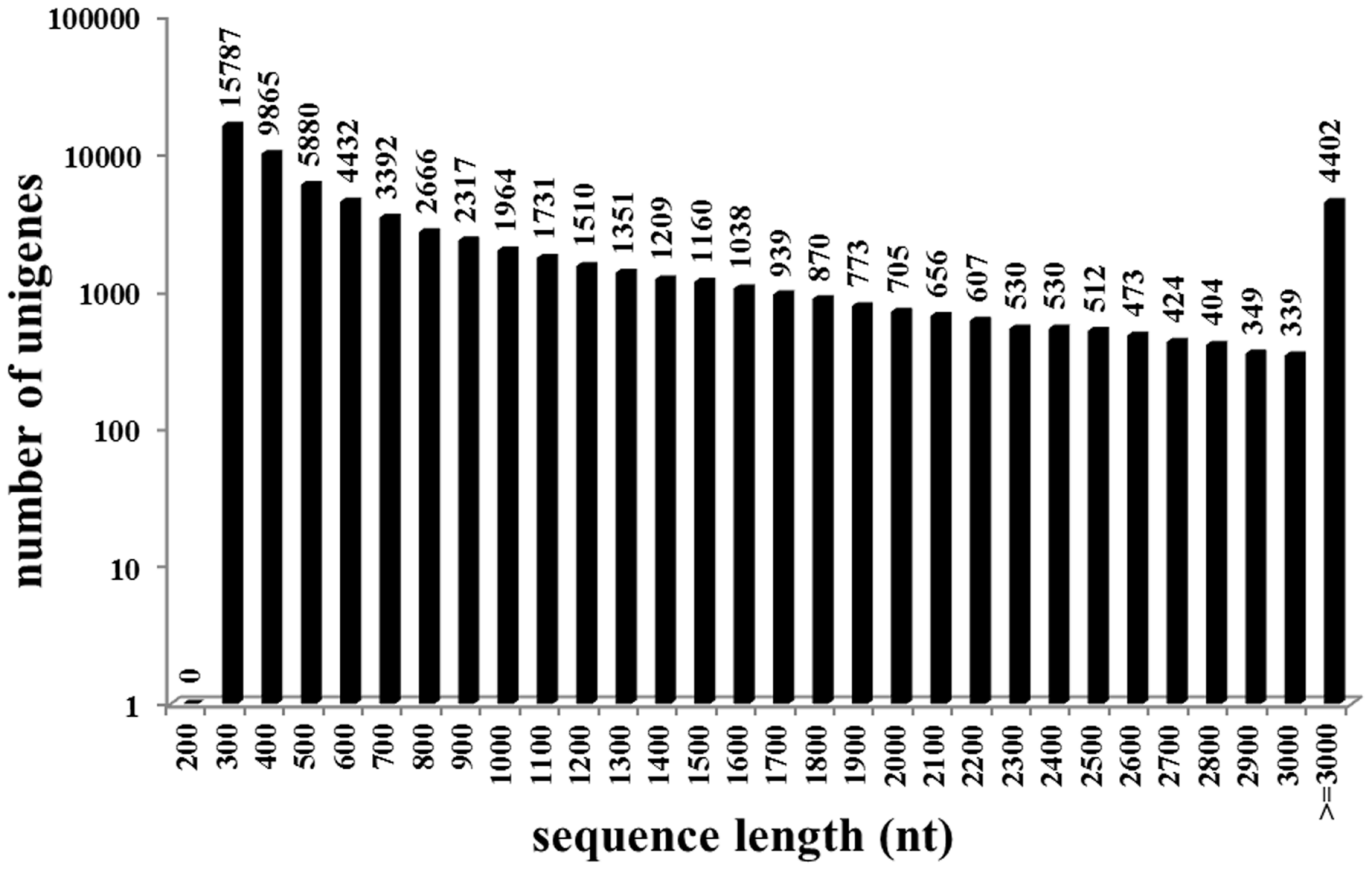
Length distribution of all-unigenes after clustering unigenes in each group. X axis represents sequence length intervals. Y axis represents the number of unigenes in each interval.

Five cDNA libraries were constructed on the basis of five RNA samples as described in the Materials and methods section. By mix 15 samples into five groups for sequencing, it would be more comprehensive for depicting the transcriptome profiles during development and more targeted for comparison. Using Illumina HiSeq 2000, a total of 55,895,400, 58,816,588, 59,721,370, 59,760,506, 57,889,382 raw reads were obtained respectively. After removing adaptors and trimming low quality reads, 51,568,556 (92%), 52,824,674 (90%), 53,430,302 (89%), 53,902,786 (90%), 51,574056 (89%) clean reads were obtained, and these clean reads were assembled respectively and then clustered into 66,815 unigenes (Table 1). These data were deposited to Sequence Read Archive database of National Center for Biotechnology Information with accession numbers of SRR1460493, SRR1460494, SRR1460495, SRR1460504 and SRR1460505. The all-unigenes, totaling to 68 Mbp, with an average length of 1027 bp and N50 length of 1851 bp, were then used as references for annotation and expression analysis. The size distribution of all-unigenes was shown in Figure 2.

**Table 1:** Summary of sequencing and assembly of the transcriptome from *L. vannamei*.

Samples	Raw Reads	Clean Reads	Unigene number	Unigene Average Length	Unigene N50
E	55,895,400	51,568,556	53,822	747	1404
N	58,816,588	52,824,674	58,048	735	1314
Z	59,721,370	53,430,302	64,443	703	1219
M	59,760,506	53,902,786	66,215	705	1226
P	57,889,382	51,574,056	64,528	699	1204
All	292,083,246	263,300,374	66,815	1027	1851

RNA-Seq is a sequencing based method that allows the entire transcripts to be surveyed in a very high-throughput and quantitative manner. It has clear advantages over other approaches and is expected to revolutionize the manner in which eukaryotic transcriptomes are analyzed [14]. By sequencing five different samples individually, we built the first gene expression profiles of *L. vannamei* during early development. The average length of unigenes in each group was around 700 bp. By clustering the unigenes together into all-unigenes, we get a significantly improved assembly result with an average length 1027 bp and total length 68 Mbp. By blast search, a large number of genes which have not been reported in penaeid shrimp before were annotated and many of them play key roles in early development. These results would contribute to both the penaeid transcriptome database and comparative analysis of gene expression profiles.

### Functional annotation and classification

**Figure 3:**
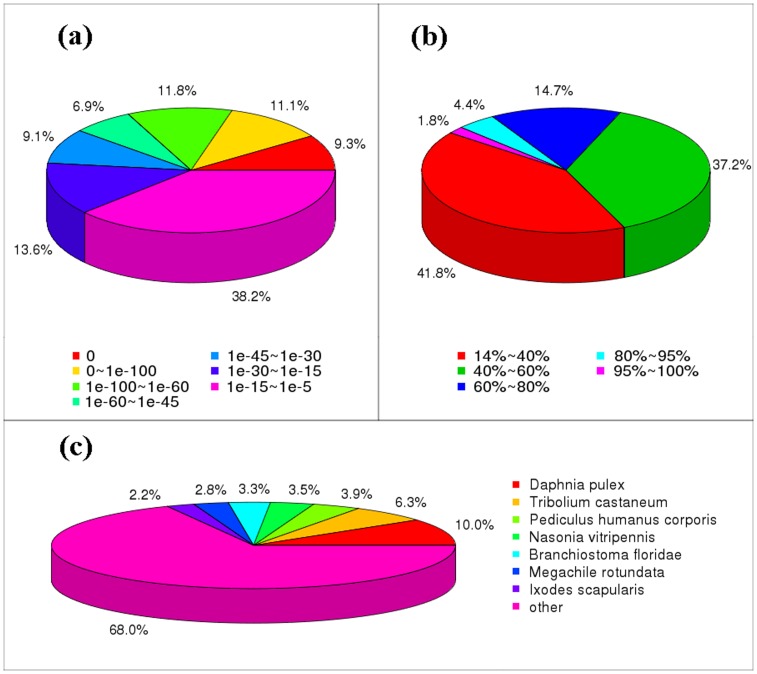
Summary of homology search of all-unigenes against nr database. (a) E-value distribution of the best blast hits; (b) Similarity distribution of the best blast hits; (c) Species distribution of the best blast hits.

In order to annotate the all-unigenes, blast alignment against the nr, nt, and Swiss-Prot databases was performed. By blast searching with a cutoff E-value < 1e-5, 32,398 (48.5%) unigenes found putative homologues in the nr protein database from NCBI, 19,363 (29.0%) unigenes found putative homologues in the nt database and 29,022 (43.4%) unigenes found putative homologues in the Swiss-Prot database. The best aligning results are used for analysis. The E-value and similarity distribution of the best blast hits for unigenes were shown in Figure 3. The distribution of best hits over species for nr annotation was also analyzed. *Daphnia pulex* (10.0%), *Tribolium castaneum* (6.3%) and *Pediculus humanus corporis* (3.9%) possess the top three maximum unigene numbers with nr annotation.

**Figure 4:**
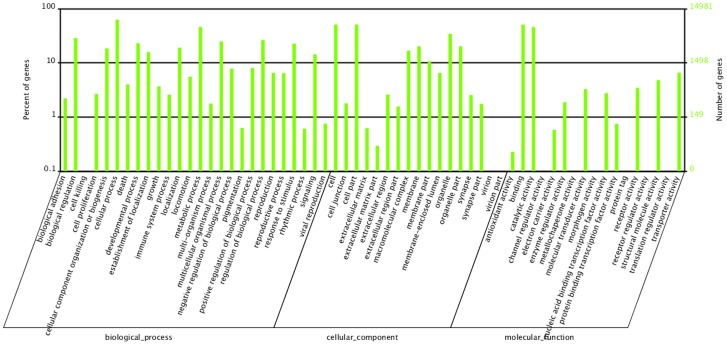
GO annotation of all-unigenes. Unigenes with GO annotation were divided into three major categories: biological process, cellular component and molecular function.

**Figure 5:**
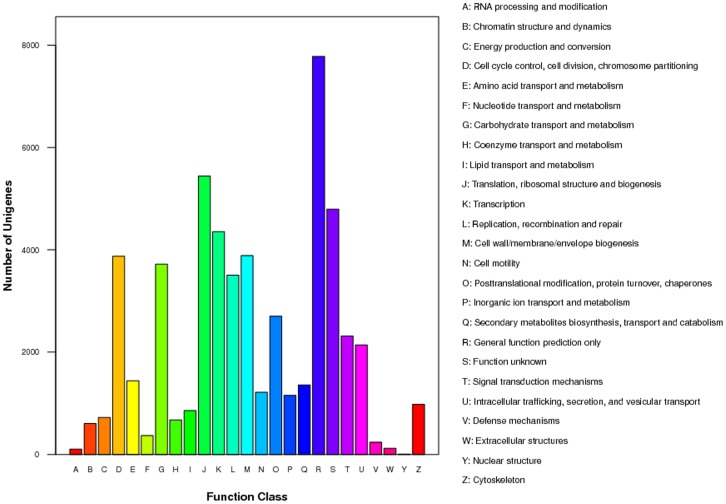
COG classification of all-unigenes. Unigenes were classified into 25 function classes. The columns represents the number of unigenes in each class.

Gene Ontology (GO) is an international standardized gene functional classification system which offers a dynamic-updated vocabulary to comprehensively describe properties of genes and their products in any organism [29]. With nr annotation, we used Blast2GO program to get GO annotation of all-unigenes. 14,981 (22.4%) were classified into diverse functional categories by GO annotation. Among them, 11,065 were mapped to biological processes including 3449 involved in development process and 551 involved in growth, 8678 were mapped to cellular components and 12,057 were mapped to molecular functions (Figure 4). Clusters of Orthologous Groups (COG) database [30] is also an important classification system for functional annotation. As for COG classification, 15,467 (23.1%) were classified into 25 functional categories (Figure 5). The largest group was “general function predicted only”, followed by “translation, ribosomal structure and biogenesis” and “function unknown”. “cell cycle control, cell division, chromosome partitioning”, containing plenty of developmental related genes, also represented a large group with 3877 unigenes.

KEGG is a database to analyze gene product during metabolism process and related gene functions in the cellular processes [31]. To identify the biological pathways involved in early development of *L. vannamei*, the KEGG pathway annotation were obtained by blast all-unigenes with KEGG database, and 26,257 (39.3%) were associated with 255 pathways including a lot of development related pathways, such as Wnt [32] (341 unigenes), Hedgehog [33] (262 unigenes), Notch [34] (170 unigenes) and so on (Table 2). The main nodes in these pathways were identified and some of them were listed in Table 2.

**Table 2:** Development related pathways and annotated key nodes.

KEGG pathway	Unigene number	Partial unigene annotation
MAPK signaling pathway	532	*EGF, EGFR, Grb2, Sos, Ras, NF1, p38, ERK5,*
Dorso-ventral axis formation	399	*Grk, Argos, Top, Drk, Egh*
Wnt signaling pathway	341	*wnt-1, wif-1, wnt-5, Frizzled, beta-catenin, GSK3, APC*
Hedgehog signaling pathway	262	*Hgdgehog, Gas1, ptc, smo, Fu, Ci, PKA, Slimb*
TGF-beta signaling pathway	172	*BMP, BMPR, Smad, ERK, Activin, ActivinR, SARA*
Notch signaling pathway	170	*Notch, TACE, Delta, Serrate, CSL*
VEGF signaling pathway	167	*VEGFR, Paxillin, casp9, Rac, CALN, PKC, SPK, MEK*
Jak-STAT signaling pathway	140	*JAK, STAT, CytokineR, CBP, SHP1,SHP2, PI3K, AKT*

### Clustering analysis and identification of differentially expressed genes (DEGs)

**Figure 6:**
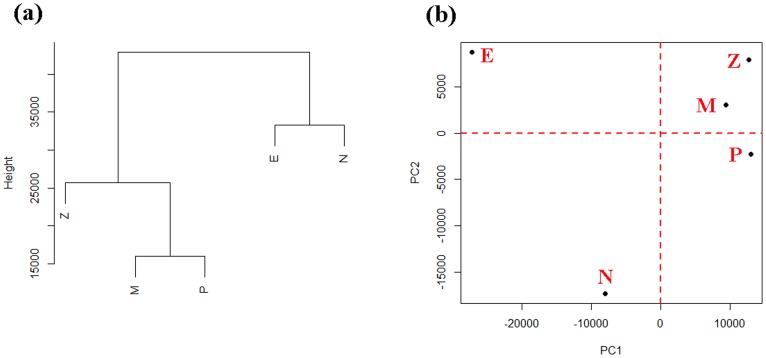
HCA and PCA plots for five samples using FPKM value of unigenes. (a) Hierarchical clustering analysis plot. The height represents the Euclidean distance; (b) Principal components analysis plot. X axis represents PC1 that explains 78.7% and Y axis represents PC2 that explains 11.2% of the total variability for gene expression.

To investigate the global transcriptional differences between stages and genes during development, hierarchical clustering analysis (HCA) and principal components analysis (PCA) were performed using whole expression datasets in each sample (Figure 6). For HCA, M and P clustered together first and then clustered with Z, while E and N clustered together eventually. For PCA plot, the first two principal components (PC1 and PC2) explained 89.9% percent of the total variability in gene expression (78.7% percent and 11.3% percent respectively). PC1 divided them into two groups: one for E and N, another for Z, M and P, which is in accordance with HCA result. The bi-dimensional plot also revealed that Z, M and P shared a relatively similar expression profile, while E and N had a relatively large difference compared to Z, M and P.

**Figure 7:**
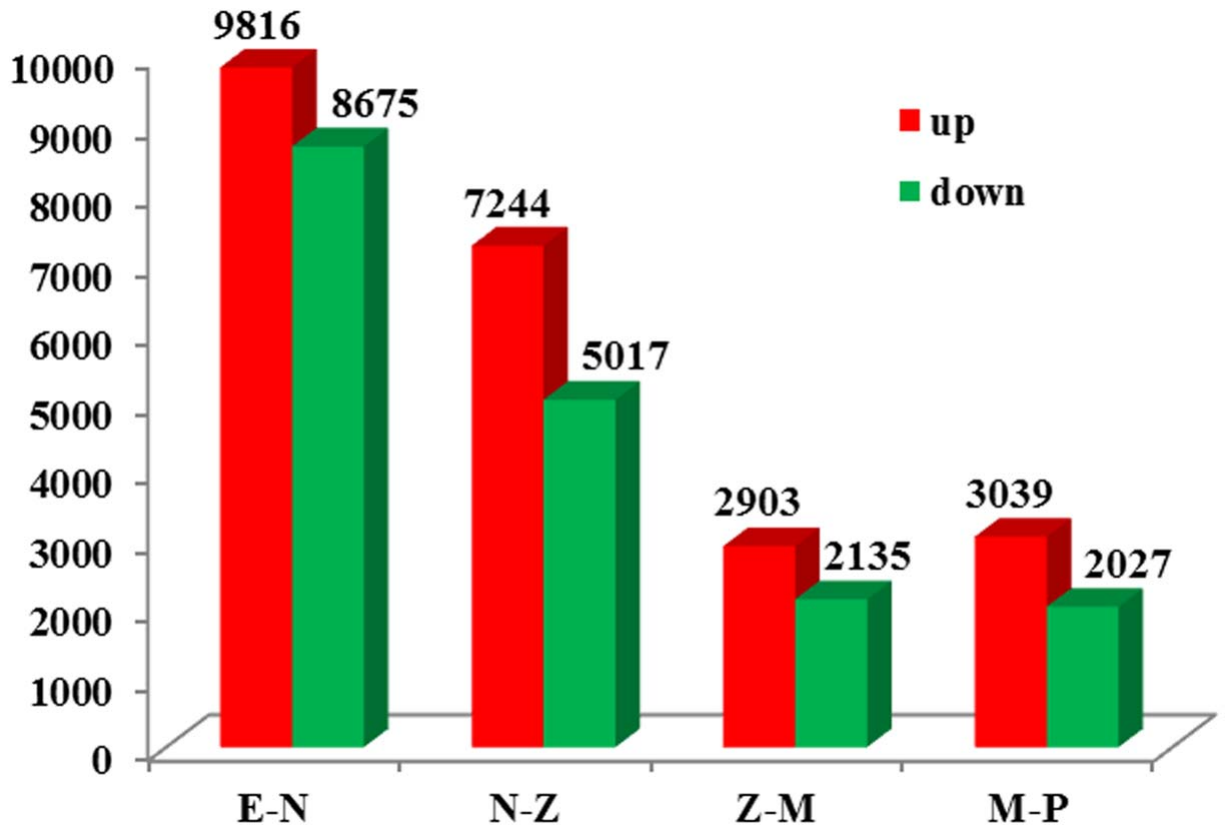
Statistics of differentially expressed unigenes between adjacent samples. Red columns represent the number of up-regulated genes while green columns represent the number of down-regulated genes.

To identify DEGs involved in early development, we use FPKM value for comparing the expression differences between adjacent samples (E-N, N-Z, Z-M and M-P). A large number of DEGs were screened with absolute value of log2ratio ≥ 1 and FDR ≤ 0.001 (Figure 7). Among 66,815 unigenes, 18,536 were identified as DEGs between E and N (9861 up-regulated, 8675 down-regulated) and 12,261 were identified as DEGs between N and Z (7244 up-regulated and 5017 down-regulated). The number of DEGs distinctly decreased when comparing Z with M (5038 DEGs with 2903 up-regulated, 2135 down-regulated) and M with P (5066 DEGs with 3039 up-regulated, 2027 down-regulated). The number of up-regulated genes was significantly more than that of down-regulated genes with a p-value 0.027 by paired t test.

The five samples could be clustered into three major groups (E for group1, N for group 2, Z, M and P for group3) according to HCA and PCA. In correspondence with this, a relatively high proportion of DEGs also occurred in E-N and N-Z, while a low proportion occurred in Z-M and M-P. These all indicated that more dramatic changes occurred in earlier transition. For E-N transition, this may related to the existence of maternal transcripts in E sample. Maternal gene products drive early development when the newly formed embryo is transcriptionally inactive [35], [36]. Embryonic transcription is initiated and many maternal RNAs are degraded until the maternal-zygotic transition [35]. For N-Z transition, the dramatic changes may relate to the higher levels of cell differentiation with organ formation in Z stage such as the formation of midgut for digestion [37] and compound eyes for locomotion [2]. In addition, the number of up-regulated genes was more than that of down-regulated genes (p = 0.027), suggesting more genes get activated along with development in order to drive further developmental events [38].

### GO term enrichment analysis and KEGG pathway enrichment analysis

GO term enrichment analysis detected significantly overrepresented GO terms in DEGs with FDR corrected p value <0.05. The top five most significantly enriched GO terms were shown in Table 3 including three ontologies: cellular component, molecular function and biological process.

**Table 3:** The top five most significantly enriched GO terms.

Compare groups	GO – Cellular Component	GO – Molecular Funciton	GO – Biological Proecss
E-N	1. myosin filament	1. microfilament motor activity	1. dorsal closure, spreading of leading edge cells
	2. myosin complex	2. myosin light chain binding	2. ecdysone-mediated induction of salivary gland cell autophagic cell death
	3. apical cortex	3. myosin binding	3. induction of programmed cell death by ecdysone
	4. cell division site	4. structural constituent of muscle	4. induction of programmed cell death by hormones
	5. cleavage furrow	5. catalytic activity	5. anterior midgut development
N-Z	1. myosin filament	1. catalytic activity	1. striated muscle myosin thick filament assembly
	2. myosin complex	2. microfilament motor activity	2. skeletal myofibril assembly
	3. apical cortex	3. myosin light chain binding	3. skeletal muscle myosin thick filament assembly
	4. striated muscle myosin thick filament	4. structural constituent of muscle	4. myosin filament organization
	5. A band	5. myosin binding	5. myosin filament assembly
Z-M	1. ribosome	1. structural molecule activity	1. translation
	2. myosin filament	2. structural constituent of ribosome	2. skeletal myofibril assembly
	3. myosin complex	3. microfilament motor activity	3. anterior midgut development
	4. actin cytoskeleton	4. myosin binding	4. myosin II filament assembly
	5. non-membrane-bounded organelle	5. myosin light chain binding	5. myosin II filament organization
M-P	1. extracellular region	1. catalytic activity	1. metabolic process
	2. myosin filament	2. peptidase activity	2. chitin metabolic process
	3. myosin complex	3. oxidoreductase activity	3. aminoglycan metabolic process
	4. ribosome	4. chitin binding	4. polysaccharide metabolic process
	5. extracellular space	5. hydrolase activity	5. carbohydrate metabolic process

The biological process ontology includes terms that represent collections of processes as well as terms that represent a specific and entire process. The enrichment of DEGs on this ontology provided a considerable perspective for understanding the biological change during early development. For E-N group, the most significant GO biological process term was “dorsal closure, spreading of leading edge cells”, followed by three GO terms involved with cell death (“ecdysone-mediated induction of salivary gland cell autophagic cell death”, “induction of programmed cell death by ecdysone” and “induction of programmed cell death by hormones”). For N-Z group, the top five most significant GO biological process terms all associated with muscle and skeletal development including “striated muscle myosin thick filament assembly”, “skeletal myofibril assembly”, “skeletal muscle myosin thick filament assembly”, “myosin filament organization” and “myosin filament assembly”. For Z-M group, the most significant GO molecular function term was “translation”, followed by “skeletal myofibril assembly”, “anterior midgut development”, “myosin II filament assembly” and “myosin II filament organization”. For M-P group, the top 5 most significant GO biological process terms all involved with metabolism process including “metabolic process”, “chitin metabolic process”, “aminoglycan metabolic process”, “polysaccharide metabolic process” and “carbohydrate metabolic process”.

To evaluate the pathways associated with DEGs, we conducted the KEGG pathway enrichment analysis. The top ten enriched pathways were listed in Table 4. Considering these top ten pathways, some appeared repeatedly in the four comparison groups like “Vibrio cholerae infection”, “Amoebiasis” and “Staphylococcus aureus infection”. At the same time, some only enriched in a specific comparison group. For example, “mRNA surveillance pathway” and “Glycosaminoglycan degradation” only appeared in E-N group, while “Hematopoietic cell lineage” and “Linoleic acid metabolism” only appeared in M-P group. For N-Z group, many metabolism related pathways were enriched including “Pancreatic secretion”, “Protein digestion and absorption”, “Amino sugar and nucleotide sugar metabolism”, “Tyrosine metabolism”, “Metabolic pathways” and “Glutathione metabolism”. For Z-M group, many enriched pathways are associated with cardiac muscle like “Viral myocarditis”, “Hypertrophic cardiomyopathy”, “Dilated cardiomyopathy” and “Cardiac muscle contraction”.

**Table 4:** The top ten enriched KEGG pathways.

	E-N	N-Z	Z-M	M-P
1	Vibrio cholerae infection	Vibrio cholerae infection	Vibrio cholerae infection	Amoebiasis
2	Amoebiasis	Amoebiasis	Ribosome	Vibrio cholerae infection
3	Viral myocarditis	Pancreatic secretion	Amoebiasis	Staphylococcus aureus infection
4	Cardiac muscle contraction	Protein digestion and absorption	Viral myocarditis	Glutathione metabolism
5	Complement and coagulation cascades	Influenza A	Hypertrophic cardiomyopathy (HCM)	Amino sugar and nucleotide sugar metabolism
6	mRNA surveillance pathway	Amino sugar and nucleotide sugar metabolism	Dilated cardiomyopathy	Renin-angiotensin system
7	Hypertrophic cardiomyopathy (HCM)	Tyrosine metabolism	Cardiac muscle contraction	Hematopoietic cell lineage
8	Protein digestion and absorption	Metabolic pathways	Influenza A	Linoleic acid metabolism
9	Renin-angiotensin system	Staphylococcus aureus infection	Amino sugar and nucleotide sugar metabolism	Metabolic pathways
10	Glycosaminoglycan degradation	Glutathione metabolism	Staphylococcus aureus infection	Complement and coagulation cascades

### Physiological changes during shrimp metamorphosis

The early development of *L. vannamei* gets through both embryonic stage and larval stage. This unique early developmental mode by metamorphosis implies dramatic changes and provides a unique opportunity to examine shrimp body reorganization [39]. Moreover, a better understanding of *L. vannamei* development would be greatly beneficial for breeding control and ensure the long-term viability of shrimp aquaculture [40]. The 15 samples we chose in this study covered the main stages across both embryonic and larval development. By sample pooling and comparative analysis, dynamic changes of gene expression can be revealed more distinctly.

**Figure 8:**
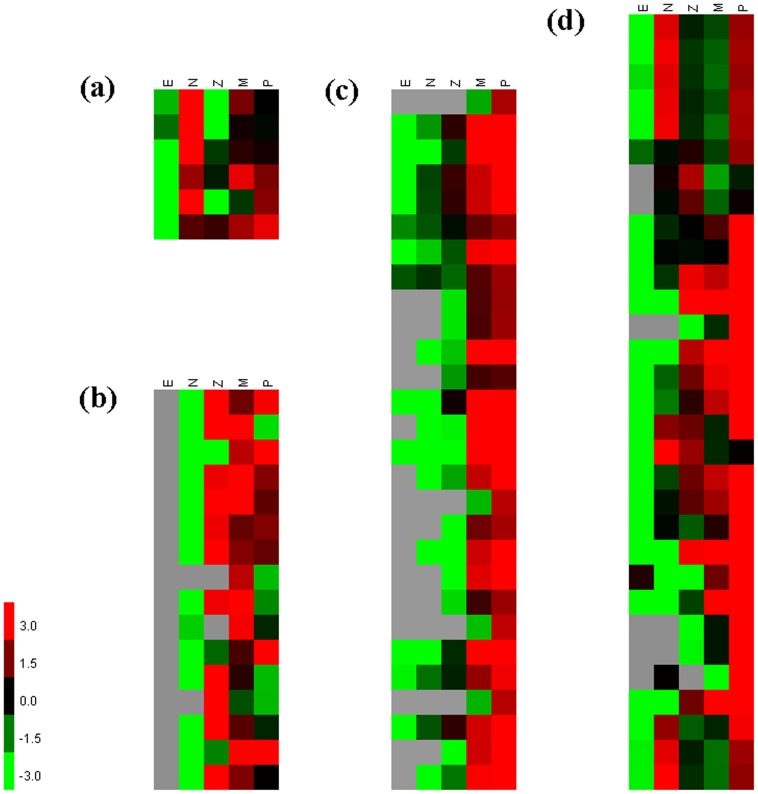
Expression profiles of genes involved in specific functions. The red-green heat maps were drawn according to FPKM value. The Gray units represent zero for FPKM value. (a) Heat map for nuclear hormone receptor E75; (b) Heat map for amylase; (c) Heat map for myosin heavy chain type a (partial); (d) Heat map for calcified cuticle protein.

GO term enrichment analysis revealed that DEGs between E and N stages were significantly enriched in hormone induced programmed cell death process which related to histolysis and reconstruction (three of five in top five most significantly enriched GO terms). Metamorphosis took place when shrimps stepped from embryo into larval development, and a series of hormones like ecdysone triggered the transition during metamorphosis and initiated programmed cell death [41]. Ecdysone signaling has been studied extensively in the larval salivary glands of Drosophila. The pulses of ecdysone regulate developmental pathways through a transcriptional hierarchy [42]: Ecdysone binds to its heterodimeric receptor which consists of ecdysone receptor (EcR) and ultraspiracle (USP). The EcR complex functions together with fushi tarazu-factor 1 (FTZ-F1) to induce transcription of the early genes including Broad Complex (BR-C), E74A, E75, and E93. The early genes then activate transcription of many late genes involved in signaling, cellular organization, apoptosis, and autophagy [43]. We identified EcR, RXR (retinoid X receptor, homolog of USP), FTZ-F1 and E75 in our dataset (Table 5) and many of them were up-regulated in E-N transition such as E75 (Figure 8), implicating the hormone signal hierarchy also existed in shrimp metamorphosis and the hormones might also act as a primary signal for programmed cell death in shrimp larval stage. Hence we infer that the ecdysone signals might have distinctly different roles between embryo and larvae.

**Table 5:** Partial functional categories and genes involved in early development of *L. vannamei*.

Functional categories	Functional genes	Number of hits	Up-regulated hits	Up-regulated group
Ecdysone signal transduction	*EcR*	3	1	E-N
	*RXR*	2	0	
	*E75*	6	6	
	*FTZ-F1*	2	2	
digestive enzyme secretion	*trypsin*	29	21	N-Z
	*chymotrypsin*	17	9	
	*amylase*	16	11	
myosin II filament assembly	*Distal-les*	4	0	Z-M
	*Ladybird*	1	0	
	*myosin heavy chain type a*	100	28	
	*myosin heavy chain type b*	77	18	
exoskeleton reconstruction	*cuticle protein*	188	125	M-P
	*calcified cuticle protein*	31	27	
	*early cuticle protein*	24	20	
	*calcification associated soluble matrix protein*	11	11	
	*calcification-associated peptide-1*	11	10	
	*crustocalcin*	2	2	

KEGG pathway enrichment analysis revealed that DEGs between N and Z stages were significantly enriched in digestive enzymes secretion and metabolic pathways (six of ten in top ten most significantly enriched KEGG pathways), indicating that source of nutrition was transforming greatly in this period. There was no feeding process in embryo and nauplius stage, and all of the nutrients come from the reservation of yolk. When shrimp became zoea, they started to eat unicellular algae or plant debris [44], [45]. Corresponding to the initiation of feeding, the expression level of many digestive enzymes increased sharply during N-Z transition such as trypsin, chymotrypsin and amylase (Table 5). Take amylase as an example, 16 unigenes were identified as alpha amylase and their expression levels all increased sharply from nauplius stage to zoea stage, and then kept relatively stable until mysis and postlarvae stage (Figure 8). The explosion of digestive enzymes in N-Z transition is consistent to larval feeding habits and also could be a symbol of a developed digestive system [44].

DEGs between Z and M stages were significantly enriched in myosin II filament assembly and organization which related to muscle development, indicating the enhancement of motor ability in this period. The prominent morphological change in Z-M transition is the appendage formation. As larvae progress to later stages, more posterior appendages are used for locomotion [46]. Nauplius and zoea use cephalic propulsion. Mysis swim using the pereopods, while postlarvae use the pleopods [47]. Therefore, we inferred that the muscle also entered a rapid growth period along with new appendage formation. Myosin II, which composed of two myosin heavy chain (MYH) subunits and four myosin light chain subunits [48], is a major component of thick filaments in muscle. For MYH, type a and type b are primarily expressed in body-wall muscle [49]. As for appendicular myogenesis, Distal-less gene marks the initiation of appendage development [50] and ladybird genes were also essential to generate a specialized type of appendage adapted for locomotion [51]. We identified these two key genes in our dataset (Table 5), and pathways which activate muscle development were also well-annotated including Wnt [52] and Notch [53] (Table 2). A large group for MYH type a and type b genes were identified in our dataset (Table 5), and the up-regulated genes of MYH type a and type b in M stage implied the rapid growth for muscles (Figure 8).

DEGs between M and P stages were significantly enriched in chitin metabolism, which related to exoskeleton reconstruction. The body surface of arthropods is covered by an extracellular material called the exoskeleton (cuticle). The exoskeleton is an assembly of chitin and cuticle proteins. Its physical properties are determined largely by the proteins it contains, and vary widely with developmental stages and body regions [54]. Chitin is the major component for exoskeleton of penaeid shrimp [55], while cuticular proteins enhance the hardness of exoskeleton in order to protect the body from predation [56]. The variation of chitin and polysaccharide metabolism during this transition suggested the reconstruction for exoskeleton. Intriguingly, cuticle proteins involved in calcification (calcified cuticle protein and early cuticle protein) were sharply up-regulated in M-P transition (Figure 8). Meanwhile, other calcification related proteins (calcification associated soluble matrix protein, calcification-associated peptide-1 and crustocalcin) were also up-regulated (Table 5). Considering larvae moved to lower water layer during M-P transition, the reinforcement of exoskeleton by calcification might be an adaptation for the transition from a planktonic life to a benthic life. Total of 188 unigenes were annotated as cuticle proteins in our dataset. They possessed a high diversity and varied in expression patterns. The cuticular protein genes were also diversified among insects: 101 cuticular protein genes have been identified in the genome of *Drosophila melanogaster* [57] and 156 in *Anopheles gambiae* [58]. The diversity of cuticle protein in transcriptional level of shrimp might contribute to the rapid generation of exoskeletons with different physical properties in different developmental stages.

**Figure 9:**
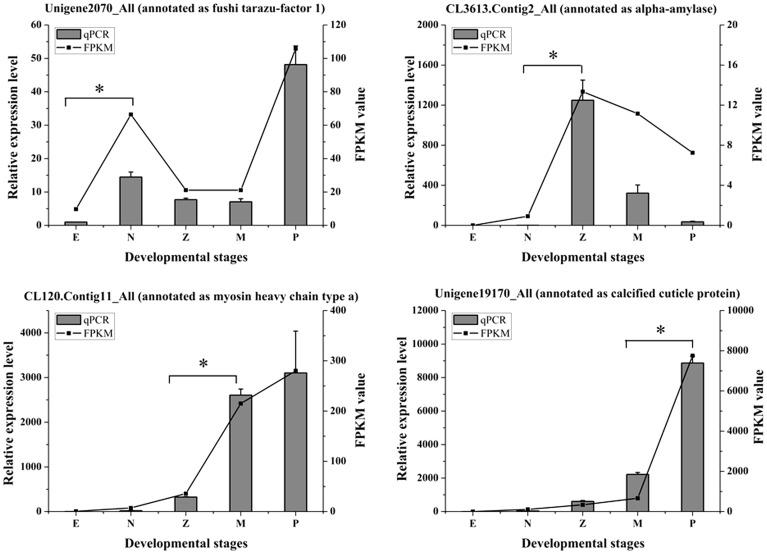
Expression profiles of four unigenes. X axis represents the developmental stages. Columns and bars represent the means and standard error of relative expression levels from qPCR result (Y axis at left). Lines represent the FPKM value from transcriptome result (Y axis at right). Asterisks represent the up-regulated transitions of each unigene.

To validate our sequencing data, four analyzed differentially expressed genes (fushi tarazu-factor 1, alpha-amylase, myosin heavy chain type a and calcified cuticle protein) were selected for quantitative real-time PCR (qPCR) analysis. The information of primers was shown in Table 6. The results (figure 9) showed that the expression profiles of transcriptome data and the qPCR data were consistent. The differentially expressed genes identified by sequencing data were also obviously up-regulated in qPCR results. Unigene2070_all (annotated as fushi tarazu-factor 1) was highly expressed in nauplius and postlarvae stages, CL3613.Contig2_All (annotated as alpha-amylase) was highly expressed in zoea stage, CL120.Contig11_All (annotated as myosin heavy chain type a) was highly expressed in mysis and postlarvae stages and Unigene19170_All (annotated as calcified cuticle protein) was highly expressed in postlarvae stage.

**Table 6:** Information of primers used for real time PCR.

Amplified sequences	annotation	Sequence (5′ to 3′)	Tm (°C)
Unigene2070_All	fushi tarazu-factor 1	F: ATTGCCAACTCAACCGTCTCTAC	60
		R: GCTACACCAGGGAACAAACCA	
CL3613.Contig2_All	alpha-amylase	F: GCGAACACTACTGGATGATTGACA	61
		R: CGAAGACATTGAGGAAGCCG	
CL120.Contig11_All	myosin heavy chain type a	F: CGCCCTCTTTCTTCTCG	54
		R: ATCTGCGACGGTGCCTA	
Unigene19170_All	calcified cuticle protein	F: TCAGGAGCGGTGTAGGAGT	55
		R: GAAGAGTTCGTGCCAATCC	
18S rRNA	18S rRNA	F: TATACGCTAGTGGAGCTGGAA	55
		R: GGGGAGGTAGTGACGAAAAAT	

## Conclusions

Our study focused on the transcriptomes of five early developmental stages in *L. vannamei*, aiming for comparative analysis of physiological changes during shrimp metamorphosis. The RNA-Seq reads were assembled and clustered into 66,815 unigenes, of which 37,292 have been annotated. The five samples could be clustered into three major groups according to gene expression patterns and the differentially expressed genes between adjacent samples were also identified. By GO term enrichment analysis, KEGG pathway enrichment analysis and functional gene profiling, the physiological changes during shrimp metamorphosis could be better understood especially histogenesis, diet transition, muscle development and exoskeleton reconstruction. This is the first study that characterized the integrated transcriptome profiles during early development of penaeid shrimp. These findings will serve as significant references for shrimp developmental biology and aquaculture research.
